# The Rules of Contagion: Why Things Spread—and Why They Stop

**DOI:** 10.3201/eid2702.204255

**Published:** 2021-02

**Authors:** Valerie J. Morley

**Affiliations:** The Pennsylvania State University, University Park, Pennsylvania, USA

**Keywords:** infectious disease, mechanistic models, popular science, book review, books and media, mathematical models, contagion, transmission

In 1902, Ronald Ross received the second Nobel Prize in Physiology or Medicine for discovering that mosquito bites transmit malaria. Determined to stop the spread of the disease, Ross developed mathematical models that demonstrated a key insight: mosquito control could effectively stop the spread of malaria without eliminating all mosquitos. This early insight showed the power of mechanistic models to inform efforts to slow the spread of infectious disease.

Over a century later, digital marketers repurposed epidemiologic models to tackle a new puzzle: spreading online content. They recognized that Instagram influencers have a lot in common with superspreaders of severe acute respiratory syndrome and that memes have R_0_ values (mathematical terms that indicate how contagious an infectious disease is).

Although their goals were different, both the digital marketers and Ronald Ross turned to mathematical models to ask the same question: why do things spread, and why do they stop? This is the question that motivates Adam Kucharski’s ambitious new book The Rules of Contagion. Kucharski (Figure), an associate professor at the London School of Hygiene and Tropical Medicine, has spent his career analyzing infectious disease outbreaks. In The Rules of Contagion, Kucharski zooms out to take a sweeping look at the science of how things, from viral infections to new ideas, spread.

**Figure:**
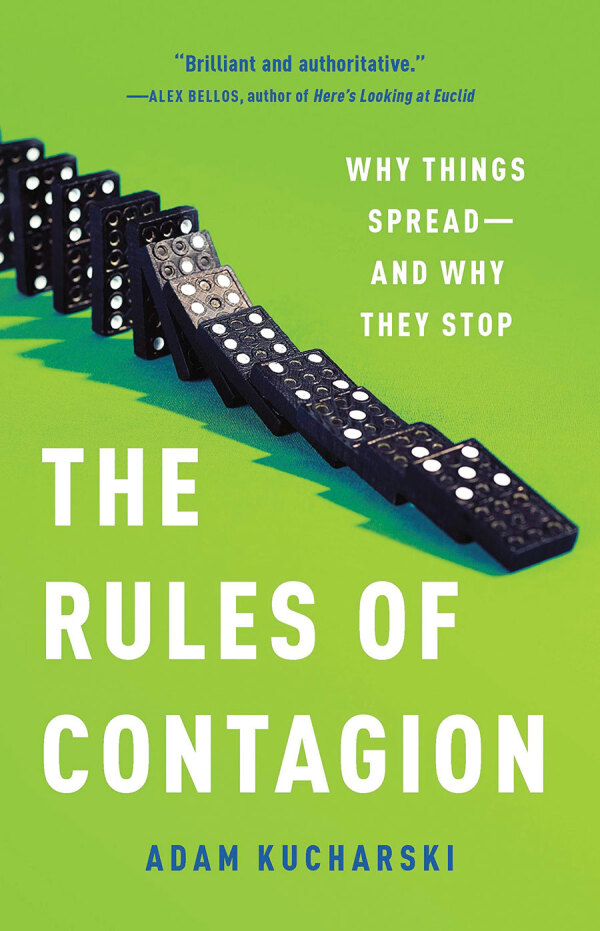
The Rules of Contagion: Why Things Spread—and Why They Stop

Kucharski artfully interweaves the science of disease outbreaks with the spread of violent crime, financial bubbles, malware attacks, and folktales. Although this book covers a lot of ground, it is an incredibly fun ride. Kucharski shows how scientists and businessmen directly apply models of infectious disease dynamics to other contexts. For example, after the 2008 financial crisis, businesses on Wall Street recruited leading theoretical biologists to forecast financial contagion, such as the spread of an economic crisis from one country to another. However, social contagion fundamentally differs from infectious disease. For instance, influenza might be transmitted by a single exposure but the spread of new ideas might depend on cumulative exposure.

Against a backdrop of pandemic and political uncertainty, this book is a timely read. Models of disease outbreaks have never been more in the public eye. The science of contagion can help societies navigate not just disease, but also pressing political issues. For example, Kucharski makes a convincing case that violent crime behaves as a contagion; by viewing violence through this lens, public health experts have offered alternatives to traditional policing. Similarly, as misinformation spreads rampantly on social media during a US election year, understanding how ideas spread online has never been more crucial.

In one whirlwind of a book, The Rules of Contagion distills lessons learned from the Zika virus epidemic, the 2008 financial crisis, the ice bucket challenge, and more. Written in clear and accessible prose, this is a rewarding read for infectious disease professionals and members of the public alike. Whether you are looking to understand the coronavirus disease pandemic or promote your ideas to the public, Kucharski will convince you that understanding contagion is essential to understanding the modern world.

